# Cost-Effectiveness Of A Workplace Ban On Sugar-Sweetened Beverage Sales: A Microsimulation Model

**DOI:** 10.1377/hlthaff.2019.01483

**Published:** 2020-07

**Authors:** Sanjay Basu, Laurie M. Jacobs, Elissa Epel, Dean Schillinger, Laura Schmidt

**Affiliations:** director of research and population health at Collective Health, in San Francisco, California, and a faculty member at the Center for Primary Care, Harvard Medical School, in Boston, Massachusetts.; researcher at the Institute for Health Policy, University of California San Francisco (UCSF), in San Francisco, California.; professor of psychiatry at UCSF.; professor of general internal medicine at UCSF.; professor of health policy in the School of Medicine and holds a joint appointment in the Philip R. Lee Institute for Health Policy Studies and the Department of Anthropology, History, and Social Medicine, all at UCSF.

## Abstract

Sugar-sweetened beverages (SSBs) increase chronic disease risk. We estimated the impact on employee health and health care spending of banning SSB sales in California-based health care organizations. We used survey data from a large, multisite health care organization in California, sampling 2,276 employees three months before and twelve months after a workplace SSB sales ban was imposed. We incorporated the survey data into a simulation model to estimate chronic disease incidence and costs. We estimated that an SSB ban as effective as the one observed would save about $300,000 per 10,000 people over ten years among similar employers, as a result of averted health care and productivity spending—after both SSB sales losses and non-SSB beverage sales gains were accounted for. Sales bans would typically need to reduce SSB consumption by 2.2 ounces per person per day for lost revenue to be fully offset if there were no increase in non-SSB beverage sales.

Sugar-sweetened beverages (SSBs), including carbonated sodas, sweetened teas, and energy drinks, are associated with elevated disease risks.^1^ Studies have shown that SSB consumption can be reduced using taxes.^2–6^ Public health commentators have also likened SSBs to tobacco products, which have been subject to complementary policies such as restrictions on purchasing to reduce consumption.^7,8^ Political will has sometimes been lacking to impose SSB taxes.^9^ An alternative approach of banning SSB sales has been instituted in schools.^10^ SSB sales bans offer a potentially more stringent limitation on sales than less universal interventions, such as limiting the number of vending machines stocked with SSBs or eliminating SSBs at one retailer,^11,12^ which have produced modest cardiometabolic effects.^13–19^

In private employer settings, SSB sales bans—insulated from industry court battles because they involve an employer’s private procurement choices—are relatively new. Recently, a group of forty-five US health care organizations voluntarily agreed to impose a workplace SSB sales ban,^20^ stopping the sale of SSBs across worksite vending machines, cafeterias, and other retail outlets.^21^ Employers may be motivated to prevent SSB-related health problems that affect productivity, recidivism, and health care costs, particularly when they sponsor employee health care coverage.^22^

Recently, an SSB sales ban was prospectively evaluated at a large multisite health care organization, the University of California San Francisco (UCSF), which has about 24,000 employees.^23^ A survey of SSB consumption by 2,276 employees three months before, versus twelve months after, the sales ban was implemented in October 2015 suggested that there had been a mean SSB consumption decrease from 11.7 ounces to 9.6 ounces per person per day (*p* < 0:001), with the largest decreases among service workers and manual laborers.^24^ A substudy of 214 employees evaluated by physical examination revealed declines in waist circumference.^23^

Our objective was to study the cost-effectiveness of an SSB workplace sales ban under a variety of consumption and cost scenarios, using data from the observational survey study at UCSF. Evidence on the ban’s effectiveness is preliminary and limited, but employers and policy makers may be interested in gaining an understanding of the magnitude of potential savings associated with modest reductions in consumption stemming from employer-driven efforts. Hence, we additionally performed a theoretical modeling exercise to identify the degree to which an SSB ban would need to be effective—in terms of reducing discounted future health care costs—to neutralize any reduction in immediate revenue for the employer from lost SSB sales.

## Study Data And Methods

The study involved a three-step simulation: drawing from the distribution of baseline SSB consumption from dietary recall data to simulate the employee populations of California health care organizations; applying the UCSF survey-based estimates of self-reported SSB consumption reduction associated with the SSB sales ban to simulate reduction from baseline SSB consumption; and using previously validated estimates for the change in risk for several chronic diseases linked to SSB consumption. These three steps constituted a cost-effectiveness analysis that computed quality-adjusted life-years (QALYs) gained and dollars saved due to an SSB workplace sales ban. Our methods followed the Consolidated Health Economic Evaluation Reporting Standards.^25^ (For a checklist of the key analysis components fulfilling the standards, see online appendix exhibit 1.)^26^ To ensure reproducible results, data and codes were posted online.^27^

### DATA SOURCES

Input data (appendix exhibit 2)^26^ were obtained from the National Health and Nutrition Examination Survey (NHANES)^28^ for 2011–16 (5,913 respondents met the inclusion criteria listed below), the Global Burden of Disease Study for 2017,^29,30^ the Medical Expenditure Panel Survey (MEPS)^31^ for 2015 (33,893 respondents met our criteria), and a multisite beverage consumption questionnaire^32^ administered three months before and twelve months after the implementation of the UCSF SSB sales ban in 2016 (there were 2,276 respondents).^23^

NHANES data were used to define demographic characteristics (age, sex, race/ethnicity, and occupational class), insurance coverage rates, and baseline disease prevalence (appendix exhibit 2).^26^ Global Burden of Disease Study data were used to define incidence (appendix exhibit 3) and mortality (appendix exhibit 4) by age and sex.^26^ MEPS data were used to estimate health care costs by outcome (appendix exhibit 2).^26^ Beverage consumption questionnaire data were used to estimate the change in SSB consumption after race/ethnicity and occupational class were adjusted for (the estimate was a weighted mean decrease of 1.5 ounces per person per day; 95% confidence interval: −0.7, −2.4) (appendix exhibit 2).^26^

### TARGET POPULATION AND SUBGROUPS

The model’s population was adults ages sixteen and older who were currently employed (part or full time) in California-based health care organizations. The target population was employed adults, with subgroups defined by occupational class (management, white collar, blue collar) and race/ethnicity (non-Hispanic white, non-Hispanic black, Hispanic, or Asian).

### STUDY PERSPECTIVES AND TIME HORIZONS

We adopted two perspectives. The first was the employer’s, which incorporated the portion of QALYs lost, health care costs, and productivity costs for the chronic disease outcomes linked to SSBs over a ten-year policy-planning horizon and accounted for the employer portion of health care spending and attrition from employer-sponsored plans (appendix exhibit 2).^26^ The second was a health care perspective, which incorporated additional QALYs lost and health care costs over the lifetime, including costs to employees and public payers (for example, Medicare after employees reached age sixty-five). A QALY is the number of years lived, weighted by an assessment of the quality of life so that 1 QALY refers to a year in perfect health and 0 QALYs refers to death.^33^ A ten-year horizon was chosen for the employer’s perspective because this was considered both the minimum time needed to accrue changes in the chronic disease outcomes and the upper-bound duration of evaluations of prevention policies that are part of self-insured employers’ workplace health care programs.^34^ The lifetime horizon was chosen for the health care perspective to correspond to the life-course theory of chronic disease.^35^

### COMPARATORS AND DISCOUNT RATE

We integrated QALYs gained and integrated dollars saved, both at a 3 percent annual discount rate, among the cohort of simulated employees for whom an SSB sales ban was instituted in 2020 compared to those for whom it was not.

### HEALTH OUTCOMES

Six disease outcomes were chosen based on the robustness of prior evidence for their association with SSB consumption: obesity (defined as having a body mass index [BMI] of at least 30 kg/m^2^),^36^ coronary heart disease (angina, myocardial infarction, cardiac arrest, ischemic heart disease, or heart failure),^37^ cerebrovascular accident (ischemic or hemorrhagic stroke),^38^ type 2 diabetes mellitus (hemoglobin A1cofatleast 6.5 percent, fasting plasma glucose of at least 126 mg/dL, two-hour oral glucose tolerance test result of at least 200 mg/dL, or a self-report of diagnosed diabetes or use of glucose-lowering medications),^39^ chronic kidney disease (estimated glomerular filtration rate of less than 90 mL/min,^40^ using the Chronic Kidney Disease-Epidemiology [CKD-EPI] equation),^41^ and both dental caries and periodontal disease (tooth decay of the permanent teeth or periodontitis, based on clinical attachment loss and periodontal probing depth).^42^ Costs and quality-adjusted disutility associated with obesity included the consequences of obesity not explicitly included in other obesity-related outcomes (for example, nonalcoholic fatty liver disease).

### MEASUREMENTS OF EFFECTIVENESS

The estimated effects of reduced SSB consumption on each outcome (appendix exhibit 2)^26^ were taken from the most recent meta-analysis or systematic review that quantified changes in SSB consumption and associated changes in the incident risk of each outcome in US adult cohorts. Estimates were standardized to reflect the average treatment effect of a reduction in consumption of 1.5 ounces per person per day of SSBs (including consumption both at and away from work) (see appendix exhibit 5 for survey sample demographics).^26^

### MEASUREMENT AND VALUATION OF QUALITY-ADJUSTED LIFE-YEARS

For all health outcomes other than obesity, disutility estimates were available from a survey-based assessment.^43^ For obesity, a prior review of disutility specific to BMI was adopted.^44^ It was weighted by the BMI distribution drawn from NHANES to assess overall disutility related to quality of life losses beyond those related to the aforementioned outcomes (for example, for obesity-related liver disease) (appendix exhibit 2).^26^

### CURRENCY, PRICE DATE, AND CONVERSION

All costs were updated for inflation to December 2019 US dollars using the Consumer Price Index.^45^ Health care costs (inpatient, outpatient, and prescription drug spending) per patient for 2015 were obtained from MEPS^31^ and attendant surveys for other obesity complications and dental disease detailed in appendix exhibit2.^26^ These included costs for employer-sponsored health insurance while employed and for postretirement or disability Medicare coverage. Additional costs included productivity costs for each health outcome, accounting for absenteeism, presenteeism, short- and long-term disability, and premature death^46–50^ (appendix exhibit 2).^26^

Overall changes in beverage sales were not observed at UCSF,^23^ because of compensatory increases in sales of bottled water, diet soda, and other non-SSBs—which fully offset decreased SSB sales. Hence, further sales losses were not simulated in the base-case simulation, only in sensitivity analyses.

### MODELING METHODS

We developed a microsimulation, which involves simulating individual people by repeatedly sampling from underlying data to capture the correlations between key factors (for example, demographic characteristics and disease prevalence), instead of simply modeling average rates. This approach permits the analysis of disparities while accounting for the key correlations and comorbid conditions reflected in the input data.^51^ Monte Carlo sampling from the input data sources was used to estimate uncertainty in all outcomes, as well as the associated mean and 95% confidence intervals around QALYs.

### SENSITIVITY ANALYSES

Sensitivity analyses recomputed cost-effectiveness outcomes from the employer’s ten-year perspective under the pessimistic scenario in which sales declines of SSBs are not offset by increased sales of bottled water or other beverages. When calculating the net discounted cost to employers of health care, productivity, and sales changes after an SSB ban, we incorporated the typical cost per ounce of SSBs^52^ and profits to employers versus vendors (appendix exhibit 2).^26^

We also computed how much reduction in SSB consumption would be necessary to offset all losses in SSB sales (ignoring all compensatory purchases of other products) to achieve cost-neutrality from the employer’s perspective. We reran the model at increasing levels of sales ban—related SSB reductions, calculating the corresponding lost SSB sales and discounted health care savings from chronic disease prevention to estimate their point of equivalence.

### LIMITATIONS

The accuracy of our estimates of the impact and cost-effectiveness of a workplace ban on SSB sales is limited by aspects of the input data and modeling approach. First, we could not account for potential long-term attitudinal changes that could take place as a result of a sales ban. Workplace bans on tobacco sales and use not only led to overall reductions in tobacco consumption but also contributed to broader normative shifts in the social acceptability of smoking.^53–56^ Similar changes may be observed for SSB sales bans.

Second, we assumed constancy of existing disease prevalence trends and associated health care costs, yet changes in the trajectory of chronic disease trends and health care cost inflation may occur in the future.

Third, we studied disease outcomes that have been strongly correlated to SSB intake, yet research continues concerning other long-term consequences of SSB consumption.^57^

Fourth, we incorporated results from a single multisite employer, located in a region where SSB intake before the ban was lower than in much of the country.^58^ SSB bans may have greater impact where baseline SSB intake is high. Conversely, such bans may have less impact where there is cultural opposition to them.

Finally, results from UCSF employees might not be fully generalizable to employees of other California health care organizations. To account for this, we computed how much effectiveness an employer must target for an SSB ban to be cost-neutral, and we concluded that there must be a reduction of at least 2.2 ounces of SSB intake per employee per day.

## Study Results

### BEFORE THE WORKPLACE SALES BAN

#### ▶ POPULATION CHARACTERISTICS:

The simulated employed population of 1,202,660 people in the California health care sector averaged 40.8 years old (interquartile range: 28.0, 52.0). It was 51.7 percent male, 11.4 percent non-Hispanic black, and 16.5 percent Hispanic or Latino. In addition, 18.9 percent had management or professional occupations, 55.8 percent had other white-collar (office-based) occupations, and 25.3 percent had blue-collar (labor-based) occupations (appendix exhibit 2).^26^

#### ▶ HEALTH OUTCOMES:

The simulated population had the following prevalences: 39.5 percent for obesity; 2.7 percent, coronary heart disease; 0.6 percent, history of cerebrovascular accident; 13.2 percent, diabetes mellitus; 13.1 percent, chronic kidney disease; and 95.9 percent, dental disease (appendix exhibit 2).^26^ Incidence by age and sex is detailed in appendix exhibit 3, and mortality in appendix exhibit 4.^26^ Given these conditions, a population of 10,000 people accrued 62,850 QALYs over ten years (95% CI: 62,818, 62,885), for an average of 0.63 QALYs per person per year of life lived and 205,414 QALYs over their lifetime (95% CI: 205,277, 205,592), for an average of 0.54 QALYs per person per year of life lived.

#### ▶ COST OUTCOMES:

From an employer perspective over a ten-year time horizon, the simulated population had a discounted cost of $27,850,112 per 10,000 people for the studied health outcomes (95% CI: $27,773,417, $27,930,469) (data not shown). Of these expenditures, 23.1 percent were related to obesity, 3.4 percent for coronary heart disease, 3.2 percent for cerebrovascular accidents, 13.8 percent for diabetes mellitus, 39.5 percent for chronic kidney disease, and 17.0 percent for dental disease.

From a health care perspective, over a lifetime horizon (including postretirement costs to Medicare), the simulated population had a discounted cost of $155,613,042 per 10,000 people for the studied health outcomes (95% CI: $155,196,921, $155,977,497). Of these expenditures, 12.6 percent were related to obesity, 14.2 percent for coronary heart disease, 22.2 percent for cerebrovascular accidents, 11.5 percent for diabetes mellitus, 35.5 percent for chronic kidney disease, and 4.0 percent for dental disease.

### COST-EFFECTIVENESS OF THE WORKPLACE SALES BAN

#### ▶ EMPLOYER PERSPECTIVE, TEN-YEAR TIME HORIZON:

The simulated SSB sales ban was estimated to reduce the incidence and mortality associated with obesity by 1.0 percent, coronary heart disease by 2.8 percent, history of cerebrovascular accidents by 1.3 percent, diabetes mellitus by 3.2 percent, chronic kidney disease by 2.5 percent, dental disease by 3.8 percent, and other causes of mortality by 2.1 percent.

The simulated sales ban saved 78.8 discounted QALYs per 10,000 people over ten years (exhibit 1), a gain of 0.1 percent (data not shown). Of the saved QALYs, 42.9 percent came from obesity-related disutility, 9.5 percent from coronary heart disease, 10.2 percent from cerebrovascular accidents, 8.8 percent from diabetes mellitus, 9.0 percent from chronic kidney disease, 10.5 percent from dental disease, and 9.0 percent from other causes of mortality. Between-group variations in QALYs saved are illustrated in exhibit 2.

From the employer perspective, the SSB sales ban produced overall cost savings as a result of health care and productivity expenditures averted, with discounted mean savings per 10,000 employees over ten years of $308,949 (exhibit 1). This was a 1.1 percent reduction (data not shown). Of the savings, 56.6 percent came from obesity-related expenditures, 2.0 percent from coronary heart disease, 4.1 percent from cerebrovascular accidents, 2.5 percent from diabetes mellitus, 2.6 percent from chronic kidney disease, and 32.1 percent from dental disease. Obesity and dental disease were generally of higher incidence before retirement age and had larger productivity or absenteeism costs, compared to the events that were rarer at a population level among the working-age population. Between-group variations in cost savings are illustrated in exhibit 3.

#### ▶ HEALTH CARE PERSPECTIVE, LIFETIME HORIZON:

The simulated SSB sales ban saved 1,069.0 discounted QALYs per 10,000 people over their lifetimes (exhibit 1), a gain of 0.5 percent (data not shown). Of the QALYs saved, 19.3 percent came from obesity-related health consequences, 13.3 percent from coronary heart disease, 12.3 percent from cerebrovascular accidents, 13.6 percent from diabetes mellitus, 13.7 percent from chronic kidney disease, 13.9 percent from dental disease, and 13.7 percent from other causes of mortality.

From a health care perspective, the SSB sales ban produced overall cost savings, with discounted mean savings per 10,000 people over their lifetimes of $706,014 (exhibit 1). This was a 0.5 percent reduction (data not shown). Of the savings, 33.6 percent were from obesity-related expenditures, 6.0 percent from coronary heart disease, 18.2 percent from cerebrovascular accidents, 3.9 percent from diabetes mellitus, 21.1 percent from chronic kidney disease, and 17.3 percent from dental disease.

### SENSITIVITY ANALYSES

If no beverage sales declines from decreased SSB consumption were compensated for by increased sales of bottled water or other beverages, employers would lose a discounted $460,215 in SSB sales per 10,000 employees over ten years (95% CI: $178,973, $889,749). In this scenario, sales losses to employers would still render the intervention highly cost-effective, at $1,896 per QALY saved (95% CI: −$6,036, $20,294) from the employer perspective over a ten-year time horizon.

For all lost SSB sales dollars to be fully offset by discounted health care cost savings (assuming no compensatory purchasing), an SSB ban would need to achieve a reduction in consumption of at least 2.2 ounces per person per day (95% CI: 1.0, 3.6).

## Discussion

In this simulation of a workplace ban on SSB sales applied to California-based health care employers, we estimated that an SSB ban as effective as the one observed (which reduced SSB consumption by 1.5 ounces per person per day), would save about $300,000 per 10,000 people over ten years among similar employers, as a result of averted health care and productivity expenditures. Our base-case estimates were based on observational data from before and after imposition of the ban and therefore cannot be taken as causally due to the ban itself.^59^ The study period was during a time of flat consumption in California (no secular change),^58^ and the reduction in consumption of 1.5 ounces per person per day is approximately 60 percent of the magnitude of impact observed from SSB taxes.^5,60^ To our knowledge, UCSF is the only employer to have collected and published data on the ban’s impact.^20^ Hence, we additionally calculated a benchmark estimate for effectiveness and estimated that sales bans would typically need to reduce SSB consumption by 2.2 ounces per person per day for lost revenue to be fully offset by reduced health care costs, if there were no increase in non-SSB beverage sales.

In the history of tobacco control, workplace smoking bans were instituted in parallel with tobacco taxation and other tobacco control initiatives. Similarly, SSB sales bans may play an important adjuvant role to SSB taxes and school-based SSB bans,^10^ particularly in the context of political challenges to SSB taxation. In this context, our modeling serves to inform planning and suggests that a workplace ban on SSB sales may produce meaningful reductions in the incidence of SSB-related disease and produce the environmental conditions for net cost savings for employers and society. ■

## Supplementary Material

Appendix

## Figures and Tables

**Exhibit 1:**
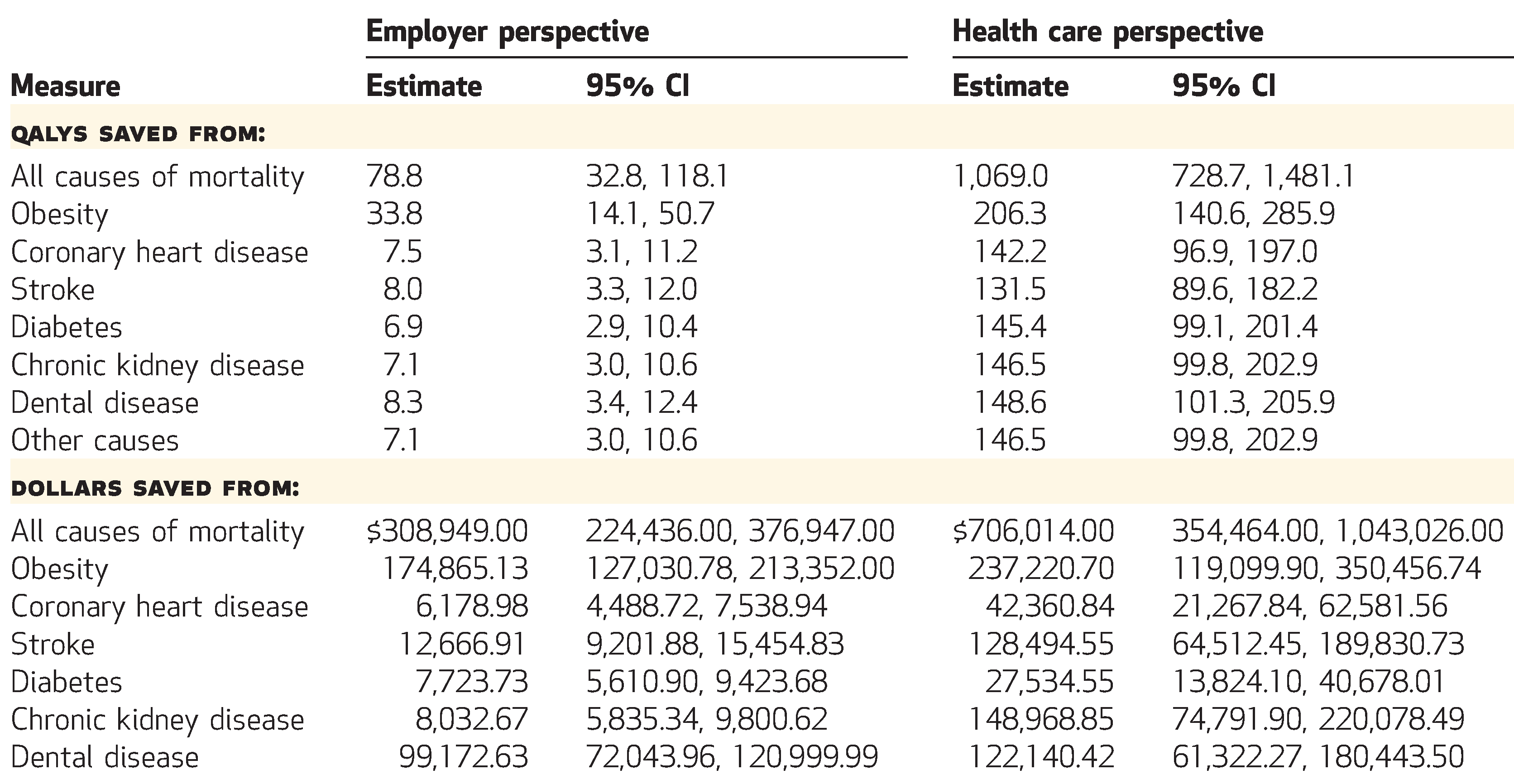
Cost-effectiveness of a simulated workplace sugar-sweetened beverage sales ban in terms of quality-adjusted life-years (QALYs) and dollars saved from employer and health care perspectives, per 10,000 employees **SOURCE** Authors analysis. **NOTES** All QALYs and dollars are discounted at a 3 percent annual rate. Employer QALYs and costs reflect a ten-year time horizon, while health care QALYs and costs reflect a lifetime horizon. Dollars are expressed in 2019 dollars adjusted for inflation using the Consumer Price Index. Rows might not sum to totals because of skewed distributions and rounding. CI is confidence interval.

**Exhibit 2:**
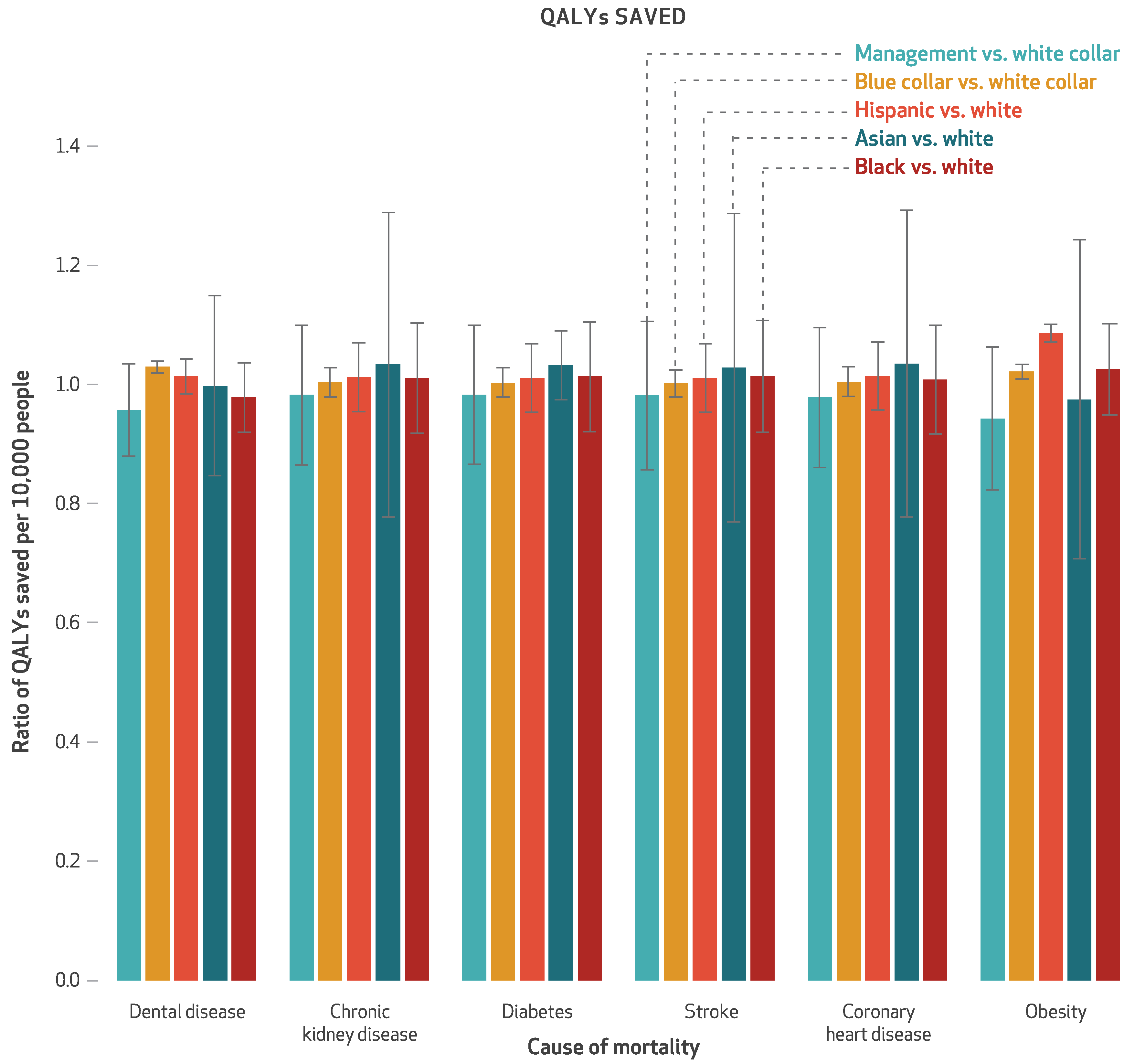
Ratios of quality-adjusted life-years (QALYs) saved per 10,000 people by the simulated workplace sales ban on sugar-sweetened beverages, by causes of mortality, race/ethnicity, and occupational class **SOURCE** Authors analysis. **NOTES** All QALYs are discounted at a 3 percent annual rate and reflect a lifetime time horizon. The error bars indicate 95% confidence intervals. Black, white, and Asian are non-Hispanic.

**Exhibit 3:**
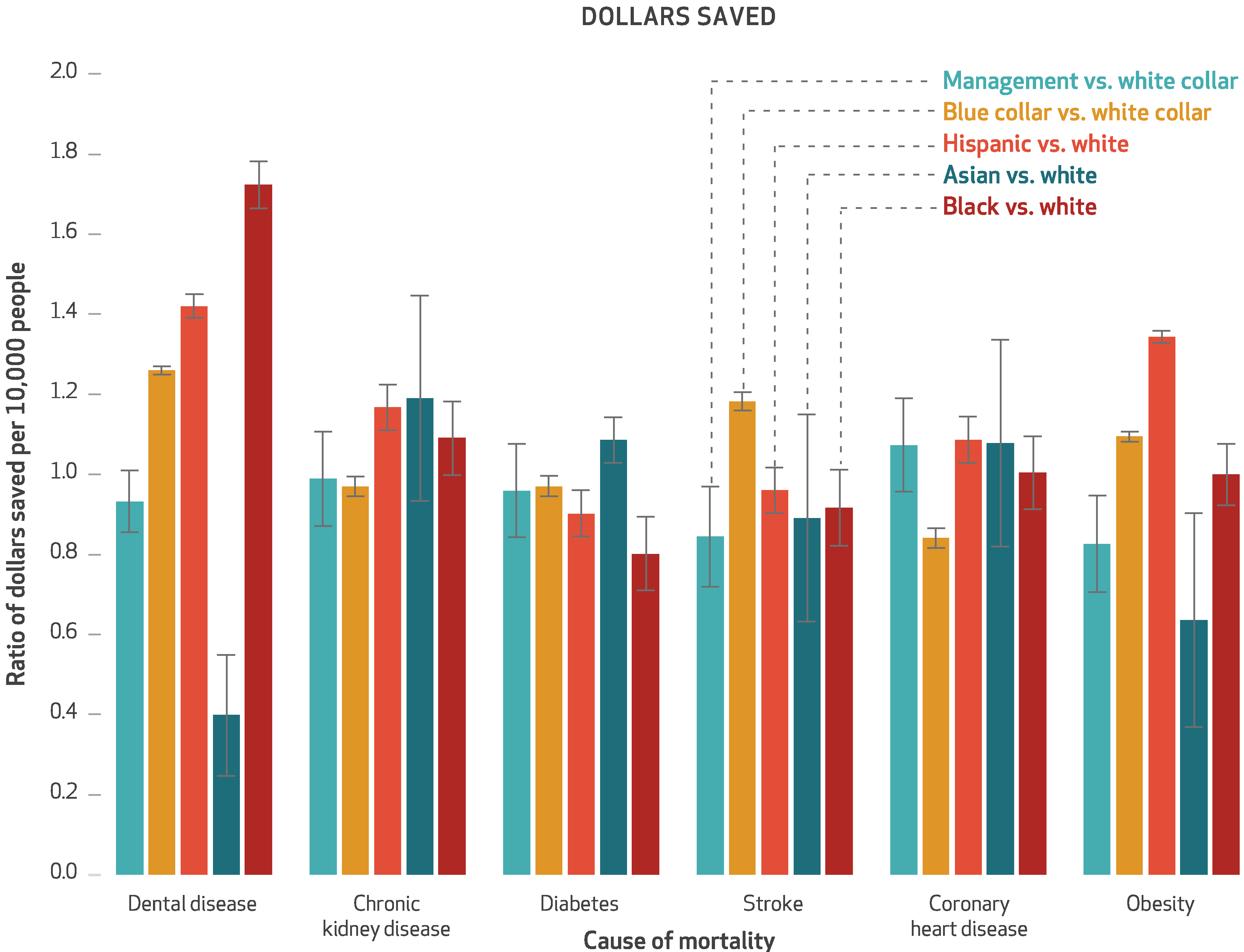
Ratios of dollars saved per 10,000 people by the simulated workplace sales ban on sugar-sweetened beverages, by causes of mortality, race/ethnicity, and occupational class **SOURCE** Authors analysis. **NOTES** All dollars are discounted at a 3 percent annual rate, reflect a lifetime time horizon, and are expressed in 2019 dollars adjusted for inflation using the Consumer Price Index. The error bars indicate 95% confidence intervals. Black, white, and Asian are non-Hispanic.

## Box

A workplace ban may produce the environmental conditions for net cost savings for employers and society.
