# Supplementary material for: Cost-Effectiveness Of A Workplace Ban On Sugar-Sweetened Beverage Sales: A Microsimulation Model
Source: Health Aff (Millwood). Author manuscript; Available in PMC 2021 Mar 17. (PMC7968436; doi:10.1377/hlthaff.2019.01483)
Supplement: Appendix [file NIHMS1674108-supplement-Appendix.pdf]

Appendix

Table of Contents

Appendix Exhibit 1. Consolidated Health Economic Evaluation Reporting Standards (CHEERS) checklist [1].

Appendix Exhibit 2. Model input data. NHANES: National Health and Nutrition Examination Survey (2011-2016) [2]; GBD: Global Burden of Disease Study (2017) [3,4]; MEPS: Medical Expenditure Panel Survey (2015) [5]. RR: relative risk. SSB: sugar-sweetened beverage. Repeated Monte Carlo sampling with replacement was performed from the uncertainty distributions listed parenthetically (Gaussian distributions build from the 95% confidence intervals listed in parentheses) to estimate uncertainty in the outcomes.

Appendix Exhibit 3. Incidence by age and sex, from the Global Burden of Disease Study (GBD, 2017) [4].

Appendix Exhibit 4. Mortality by age and sex, from the Global Burden of Disease Study (GBD, 2017) [3].

Appendix Exhibit 5. Demographic distribution of the surveyed population and association of demographic characteristics with consumption change in SSBs (2016; N = 2,276) [6].

Appendix Exhibit 6. Model diagram. NHANES: National Health and Nutrition Examination Survey (2011-2016); GBD: Global Burden of Disease Study (2017); MEPS: Medical Expenditure Panel Survey (2015). QALYs: quality-adjusted life-years. SSB: sugar-sweetened beverage.

Appendix References.

Appendix Exhibit 1. Consolidated Health Economic Evaluation Reporting Standards (CHEERS) checklist[1]

Section/item	Item No	Recommendation	Reported on
Title and abstract			
Title	1	Identify the study as an economic evaluation or use more specific terms such as “cost-effectiveness analysis”, and describe the interventions compared.	Title
Abstract	2	Provide a structured summary of objectives, perspective, setting, methods (including study design and inputs), results (including base case and uncertainty analyses), and conclusions.	Abstract
Introduction			
Background and objectives	3	Provide an explicit statement of the broader context for the study.	Intro paragraph 1
		Present the study question and its relevance for health policy or practice decisions.	Intro paragraph 4
Methods			
Target population and subgroups	4	Describe characteristics of the base case population and subgroups analyzed, including why they were chosen.	Methods paragraph 3
Setting and location	5	State relevant aspects of the system(s) in which the decision(s) need(s) to be made.	Methods paragraph 3
Study perspective	6	Describe the perspective of the study and relate this to the costs being evaluated.	Methods paragraph 4
Comparators	7	Describe the interventions or strategies being compared and state why they were chosen.	Methods paragraph 5
Time horizon	8	State the time horizon(s) over which costs and consequences are being evaluated and say why appropriate.	Methods paragraph 4
Discount rate	9	Report the choice of discount rate(s) used for costs and outcomes and say why appropriate.	Methods paragraph 5
Choice of health outcomes	10	Describe what outcomes were used as the measure(s) of benefit in the evaluation and their relevance for the type of analysis performed.	Methods paragraph 6
Measurement of effectiveness	11a	Single study-based estimates: Describe fully the design features of the single effectiveness study and why the single study was a sufficient source of clinical effectiveness data.	Methods paragraph 7
	11b	Synthesis-based estimates: Describe fully the methods used for identification of included studies and synthesis of clinical effectiveness data.	Methods paragraph 7
Measurement and valuation of preference-based outcomes	12	If applicable, describe the population and methods used to elicit preferences for outcomes.	Methods paragraph 8
Estimating resources and costs	13a	Single study-based economic evaluation: Describe approaches used to estimate resource use associated with the alternative interventions. Describe primary or secondary research methods for valuing each resource item in terms of its unit cost. Describe any adjustments made to approximate to opportunity costs.	Methods paragraph 9

	13b	Model-based economic evaluation: Describe approaches and data sources used to estimate resource use associated with model health states. Describe primary or secondary research methods for valuing each resource item in terms of its unit cost. Describe any adjustments made to approximate to opportunity costs.	Methods paragraph 9
Currency, price date, and conversion	14	Report the dates of the estimated resource quantities and unit costs. Describe methods for adjusting estimated unit costs to the year of reported costs if necessary. Describe methods for converting costs into a common currency base and the exchange rate.	Methods paragraph 9
Choice of model	15	Describe and give reasons for the specific type of decision-analytical model used. Providing a figure to show model structure is strongly recommended.	Methods paragraph 11
Assumptions	16	Describe all structural or other assumptions underpinning the decision-analytical model.	Methods paragraph 12
Analytical methods	17	Describe all analytical methods supporting the evaluation. This could include methods for dealing with skewed, missing, or censored data; extrapolation methods; methods for pooling data; approaches to validate or make adjustments (such as half cycle corrections) to a model; and methods for handling population heterogeneity and uncertainty.	Methods paragraph 13-14
Results			
Study parameters	18	Report the values, ranges, references, and, if used, probability distributions for all parameters. Report reasons or sources for distributions used to represent uncertainty where appropriate. Providing a table to show the input values is strongly recommended.	Results paragraphs 1-3, Table 1
Incremental costs and outcomes	19	For each intervention, report mean values for the main categories of estimated costs and outcomes of interest, as well as mean differences between the comparator groups. If applicable, report incremental cost-effectiveness ratios.	Results paragraph 6-10, Table 2
Characterizing uncertainty	20a	Single study-based economic evaluation: Describe the effects of sampling uncertainty for the estimated incremental cost and incremental effectiveness parameters, together with the impact of methodological assumptions (such as discount rate, study perspective).	Methods paragraph 13, Table 2
	20b	Model-based economic evaluation: Describe the effects on the results of uncertainty for all input parameters, and uncertainty related to the structure of the model and assumptions.	Methods paragraph 13, Table 2
Characterizing heterogeneity	21	If applicable, report differences in costs, outcomes, or cost-effectiveness that can be explained by variations between subgroups of patients with different baseline characteristics or other observed variability in effects that are not reducible by more information.	Results paragraph 11-12
Discussion			
Study findings, limitations, generalizability, and current knowledge	22	Summarize key study findings and describe how they support the conclusions reached. Discuss limitations and the generalizability of the findings and how the findings fit with current knowledge.	Discussion paragraphs 1-6
Other			

Source of funding	23	Describe how the study was funded and the role of the funder in the identification, design, conduct, and reporting of the analysis. Describe other non-monetary sources of support.	Funding statement
Conflicts of interest	24	Describe any potential for conflict of interest of study contributors in accordance with journal policy. In the absence of a journal policy, we recommend authors comply with International Committee of Medical Journal Editors recommendations.	Conflicts of interest statement

Notes: A QALY is the number of years lived, weighted by an assessment of the quality of life due to disease, such that 1 QALY refers to a year in perfect health and 0 QALYs to the state of death [7]. The 10-year horizon was chosen for the employer perspective because this was judged as the minimum time for accruing changes in all of the chosen chronic disease outcomes specified below, and corresponds to the upper bound duration of self-insured employer workplace healthcare program prevention policy evaluations [8]. The lifetime horizon was chosen for the healthcare perspective to correspond to a life-course theory of chronic disease and associated cost-effectiveness guidelines [9].

Appendix Exhibit 2. Model input data. 95% confidence intervals in parentheses. NHANES: National Health and Nutrition Examination Survey (2011-2016) [2]; GBD: Global Burden of Disease Study (2017) [3,4]; MEPS: Medical Expenditure Panel Survey (2015) [5]. RR: relative risk. SSB: sugar-sweetened beverage. Repeated Monte Carlo sampling was performed from the uncertainty distributions listed parenthetically (Gaussian distributions build from the 95% confidence intervals listed in parentheses) to estimate uncertainty in the outcomes.

**Parameter(s)**	**Value(s)**	**Data source**
Age, years	40.8 (interquartile range: 28.0, 52.0)	NHANES
Sex, % male	51.7%	NHANES
Race/ethnicity	11.4% non-Hispanic Black,<br>16.5% Hispanic/Latino	NHANES
Occupational class	18.9% management/professional,<br>55.8% white-collar/office-based,<br>25.3% blue-collar/labor-based	NHANES
Baseline disease prevalence	39.5% obesity,<br>2.7% coronary heart disease,<br>0.6% history of cerebrovascular accident,<br>13.2% diabetes mellitus,<br>13.1% chronic kidney disease,<br>95.9% dental disease	NHANES
Disease incidence, per 10,000 per year*	145.7 obesity,<br>74.3 coronary heart disease,<br>42.3 cerebrovascular accident,<br>52.1 diabetes mellitus,<br>9.8 chronic kidney disease,<br>2873.5 dental disease	GBD, see Online Supporting Materials Table 2 for age/sex breakdown
Mortality, per 10,000 per year	31.0 obesity,<br>45.7 coronary heart disease,<br>13.8 cerebrovascular accident,<br>4.5 diabetes mellitus,<br>7.2 chronic kidney disease,<br><1 dental disease,<br>120.9 all other causes	GBD; see Online Supporting Materials Table 3 for age/sex breakdown
Healthcare costs per outcome per year, US$2019[†]	$1814 obesity,<br>$4283 (employed) and $2220 (Medicare) coronary heart disease,<br>$7721 (employed) and $10312 (Medicare) cerebrovascular accident,<br>$2916 (employed) and $3124 (Medicare) diabetes mellitus,<br>$4857 (employed) and $5725 (Medicare) chronic kidney disease,<br>$1555 dental disease	MEPS
Productivity costs per outcome per year, US$2019	$1022 obesity,<br>$221 coronary heart disease and cerebrovascular accident,	[10–14]

	$599 diabetes mellitus,<br>$1435 chronic kidney disease,<br>$160 dental disease	
Employee attrition	23% (used in a binomial probability function to drop an employee from the simulated employer population to exclude from employer healthcare costs)	[15]
SSB consumption reduction, ounces per person per day, attributable to ban	Total (work and non-work): 1.5 (95% CI: 0.7, 2.4),<br>Total (work only): 0.7 (95% CI: 0.3, 1.3),<br>Total (non-work only): 0.8 (95% CI: 0.4, 1.2),<br>Regular soda: 0.5 (95% CI: 0.1, 0.8),<br>Sweetened fruit drinks: 0.5 (95% CI: 0.3, 0.8),<br>Sports drinks: 0.3 (95% CI: 0.1, 0.5),<br>Sweetened coffee/tea: 0.2 (95% CI: -0.4, 0.8)	[6]
Change in outcome risk, given 1.5 ounces/person/day decline in SSB consumption from ban**	0.80 weight (kg) change,<br>0.97 RR coronary heart disease,<br>0.99 RR cerebrovascular accident,<br>0.97 RR diabetes mellitus,<br>0.98 RR chronic kidney disease,<br>0.96 RR dental disease,<br>0.98 RR all-cause mortality	[16–21]
Disutility used for quality-adjusted life-year calculations	0.85 obesity,<br>0.89 coronary heart disease,<br>0.69 cerebrovascular accident,<br>0.96 diabetes mellitus,<br>0.99 chronic kidney disease,<br>0.99 dental disease	[22,23]
Proportion of SSB sales conferred as profits to employers	42%	[6]
SSB cost per 12 fluid ounce serving, $2019	$0.24 (95% CI: $0.20, $0.29)	[24]

*for obesity, linear secular trends in weight by age were used from NHANES, as GBD data were limited to annual percent changes in obesity rather than incidence.

† for obesity, costs were obtained from [25], and for dental expenditures, costs were obtained from [26], as these costs were not directly available from MEPS. Note that cost differences between employed and Medicare populations inherently include differences in severity at presentation.

** weight change is in kilograms over 3 years change from a 1.5 ounces/person/day reduction in sugar-sweetened beverage consumption. Other estimates are relative risk in incidence/mortality.

Appendix Exhibit 3. Incidence by age and sex, from the Global Burden of Disease Study (GBD, 2017) [4]. Units are cases per 10,000 people. For obesity, linear secular trends in weight change by age using NHANES data were used for incidence projection.

**Outcome**	**Age 15-49**		**Age 50-69**		**Age 70+**	
	**Male**	**Female**	**Male**	**Female**	**Male**	**Female**
Coronary heart disease	8.47	3.53	75	33.47	192.86	132.68
Cerebrovascular accident	3.95	3.99	29.36	24.39	93.21	98.7
Type 2 diabetes mellitus	33.01	25.59	115.84	88.37	29.9	19.9
Chronic kidney disease	6.37	9.87	75.73	79.6	209.61	226.48
Dental disease	4472.52	4566.26	2332.57	2459.56	1699.57	1710.43

Appendix Exhibit 4. Mortality by age and sex, from the Global Burden of Disease Study (GBD, 2017) [3]. Units are cases per 10,000 people.

**Outcome**	**Age 15-49**		**Age 50-69**		**Age 70+**	
	**Male**	**Female**	**Male**	**Female**	**Male**	**Female**
Obesity	2.00	0.98	22.45	13.04	71.13	76.30
Coronary heart disease	1.61	0.61	22.21	9.00	130.79	109.81
Cerebrovascular accident	0.40	0.33	4.53	3.23	37.06	44.07
Type 2 diabetes mellitus	0.15	0.10	2.89	1.77	12.17	9.84
Chronic kidney disease	0.26	0.18	2.01	2.09	20.91	16.91
Dental disease	0.00	0.00	0.00	0.00	0.00	0.00
Other all-cause	2.00	0.98	22.45	13.04	71.13	76.30

Appendix Exhibit 5. Demographic distribution of the surveyed population (2016; N = 2,276)[6].

**Characteristic**	**Frequency in UCSF survey population (%)**
**Sex**	
Female	64.2%
**Race/ethnicity**	
White	32.1%
Black/African-American	7.7%
Asian-American	32.3%
Hispanic/Latino/a	17.6%
**Occupational class**	
Medical technicians	11.6%
Support, clerks, analysts	23.6%
Service, maintenance, police	16.7%
Miscellaneous and technical	30.6%
Medical	9.1%
Academic	8.4%

Appendix Exhibit 6. Model diagram. NHANES: National Health and Nutrition Examination Survey (2011-2016); GBD: Global Burden of Disease Study (2017); MEPS: Medical Expenditure Panel Survey (2015). QALYs: quality-adjusted life-years. SSB: sugar-sweetened beverage.

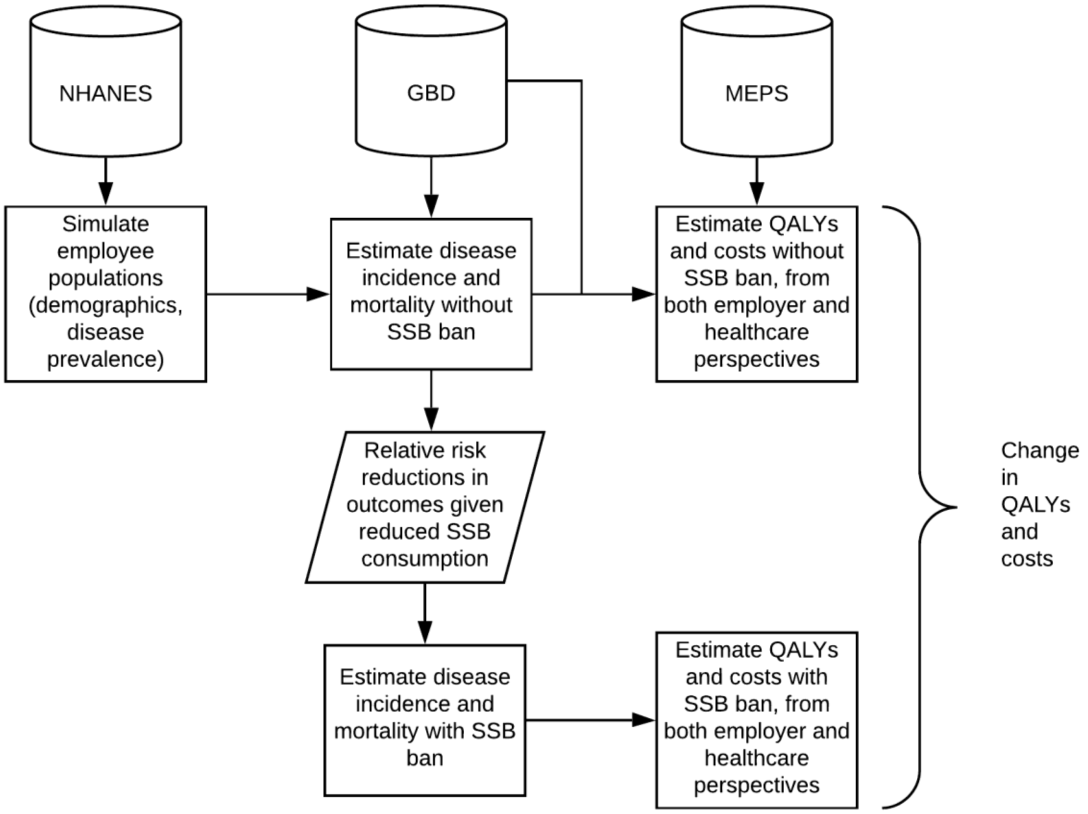

Appendix References

1. Husereau D, Drummond M, Petrou S, Carswell C, Moher D, Greenberg D, et al. Consolidated Health Economic Evaluation Reporting Standards (CHEERS) statement. Cost Eff Resour Alloc. 2013;11(1):6.
2. CDC/National Center for Health Statistics. National Health and Nutrition Examination Survey (NHANES). Atlanta; 2017.
3. GBD 2017 Causes of Death Collaborators GA, Abate D, Abate KH, Abay SM, Abbafati C, Abbasi N, et al. Global, regional, and national age-sex-specific mortality for 282 causes of death in 195 countries and territories, 1980-2017: a systematic analysis for the Global Burden of Disease Study 2017. Lancet (London, England) [Internet]. 2018 Nov 10 [cited 2019 Jun 5];392(10159):1736–88. Available from: http://www.ncbi.nlm.nih.gov/pubmed/30496103
4. GBD 2017 Disease and Injury Incidence and Prevalence Collaborators SL, Abate D, Abate KH, Abay SM, Abbafati C, Abbasi N, et al. Global, regional, and national incidence, prevalence, and years lived with disability for 354 diseases and injuries for 195 countries and territories, 1990-2017: a systematic analysis for the Global Burden of Disease Study 2017. Lancet (London, England) [Internet]. 2018 Nov 10 [cited 2019 Jun 5];392(10159):1789–858. Available from: http://www.ncbi.nlm.nih.gov/pubmed/30496104
5. Agency for Healthcare Research and Quality. Medical Expenditure Panel Survey. Rockville; 2015.
6. Epel ES, Hartman A, Jacobs L, Leung C, Cohn MA, Jensen L, et al. Association of a Workplace Sales Ban on Sugar-Sweetened Beverages With Employee Consumption of Sugar-Sweetened Beverages and Health. JAMA Intern Med [Internet]. 2019;Published online October 28, 2019. Available from: doi:https://doi-org.iclibezp1.cc.ic.ac.uk/10.1001/jamainternmed.2019.4434
7. Sassi F. Calculating QALYs, comparing QALY and DALY calculations. Health Policy Plan. 2006;21(5):402–8.
8. Centers for Disease Control and Prevention. Workplace Health in America 2017. Atlanta; 2018.
9. Sanders GD, Neumann PJ, Basu A, Brock DW, Feeny D, Krahn M, et al. Recommendations for Conduct, Methodological Practices, and Reporting of Cost-effectiveness Analyses. JAMA [Internet]. 2016 Sep 13 [cited 2019 Jun 5];316(10):1093. Available from: http://jama.jamanetwork.com/article.aspx?doi=10.1001/jama.2016.12195
10. Song X, Quek RGW, Gandra SR, Cappell KA, Fowler R, Cong Z. Productivity loss and indirect costs associated with cardiovascular events and related clinical procedures. BMC Health Serv Res [Internet]. 2015 Dec 25 [cited 2019 Jun 6];15(1):245. Available from: https://bmchealthservres.biomedcentral.com/articles/10.1186/s12913-015-0925-x
11. de Vries EF, Rabelink TJ, van den Hout WB. Modelling the Cost-Effectiveness of Delaying End-Stage Renal Disease. Nephron [Internet]. 2016 [cited 2019 Jun 6];133(2):89–97. Available from: http://www.ncbi.nlm.nih.gov/pubmed/27270045
12. Goettler A, Grosse A, Sonntag D. Productivity loss due to overweight and obesity: a systematic review of indirect costs. BMJ Open [Internet]. 2017 Oct 5 [cited 2019 Jun

6];7(10):e014632. Available from: http://www.ncbi.nlm.nih.gov/pubmed/28982806
13. Righolt AJ, Jevdjevic M, Marcenes W, Listl S. Global-, Regional-, and Country-Level Economic Impacts of Dental Diseases in 2015. J Dent Res [Internet]. 2018 May 17 [cited 2019 Jun 6];97(5):501–7. Available from: http://journals.sagepub.com/doi/10.1177/0022034517750572
14. Ramsey S, Summers KH, Leong SA, Birnbaum HG, Kemner JE, Greenberg P. Productivity and medical costs of diabetes in a large employer population. Diabetes Care [Internet]. 2002 Jan 1 [cited 2019 Jun 6];25(1):23–9. Available from: http://www.ncbi.nlm.nih.gov/pubmed/11772896
15. Mercer. North American Employee Turnover: Trends & Effects [Internet]. 2018 [cited 2019 Jun 13]. Available from: https://www.imercer.com/content/article/employee-turnover.aspx
16. Rebholz CM, Young BA, Katz R, Tucker KL, Carithers TC, Norwood AF, et al. Patterns of Beverages Consumed and Risk of Incident Kidney Disease. Clin J Am Soc Nephrol [Internet]. 2019 Jan 7 [cited 2019 Jun 13];14(1):49–56. Available from: http://www.ncbi.nlm.nih.gov/pubmed/30591520
17. Imamura F, O'Connor L, Ye Z, Mursu J, Hayashino Y, Bhupathiraju SN, et al. Consumption of sugar sweetened beverages, artificially sweetened beverages, and fruit juice and incidence of type 2 diabetes: systematic review, meta-analysis, and estimation of population attributable fraction. BMJ [Internet]. 2015 Jul 21 [cited 2019 Jun 13];351:h3576. Available from: http://www.ncbi.nlm.nih.gov/pubmed/26199070
18. Bernabé E, Vehkalahti MM, Sheiham A, Aromaa A, Suominen AL. Sugar-sweetened beverages and dental caries in adults: A 4-year prospective study. J Dent [Internet]. 2014 Aug [cited 2019 Jun 13];42(8):952–8. Available from: http://www.ncbi.nlm.nih.gov/pubmed/24813370
19. Malik VS, Li Y, Pan A, De Koning L, Schernhammer E, Willett WC, et al. Long-Term Consumption of Sugar-Sweetened and Artificially Sweetened Beverages and Risk of Mortality in US Adults. Circulation [Internet]. 2019 Apr 30 [cited 2019 Jun 13];139(18):2113–25. Available from: https://www.ahajournals.org/doi/10.1161/CIRCULATIONAHA.118.037401
20. Hall KD, Sacks G, Chandramohan D, Chow CC, Wang YC, Gortmaker SL, et al. Quantification of the effect of energy imbalance on bodyweight. Lancet [Internet]. 2011 Aug 27 [cited 2019 Jun 13];378(9793):826–37. Available from: http://www.ncbi.nlm.nih.gov/pubmed/21872751
21. Wang YC, Coxson P, Shen Y-M, Goldman L, Bibbins-Domingo K. A Penny-Per-Ounce Tax On Sugar-Sweetened Beverages Would Cut Health And Cost Burdens Of Diabetes. Health Aff [Internet]. 2012 Jan 2 [cited 2019 Jun 13];31(1):199–207. Available from: http://www.healthaffairs.org/doi/10.1377/hlthaff.2011.0410
22. Salomon JA, Haagsma JA, Davis A, de Noordhout CM, Polinder S, Havelaar AH, et al. Disability weights for the Global Burden of Disease 2013 study. Lancet Glob Heal [Internet]. 2015 Nov 1 [cited 2019 Jun 5];3(11):e712–23. Available from: https://linkinghub.elsevier.com/retrieve/pii/S2214109X15000698
23. Craig BM, Tseng DS. Cost-effectiveness of gastric bypass for severe obesity. Am J Med [Internet]. 2002 Oct 15 [cited 2019 Jun 5];113(6):491–8. Available from:

http://www.ncbi.nlm.nih.gov/pubmed/12427499
24. Kern DM, Auchincloss AH, Ballester LS, Robinson LF. Neighbourhood variation in the price of soda relative to milk and its association with neighbourhood socio-economic status and race. Public Health Nutr [Internet]. 2016 Dec 30 [cited 2019 Jun 12];19(18):3386–96. Available from: https://www.cambridge.org/core/product/identifier/S1368980016001579/type/journal_article
25. Finkelstein EA, Trogdon JG, Cohen JW, Dietz W. Annual Medical Spending Attributable To Obesity: Payer-And Service-Specific Estimates. Health Aff [Internet]. 2009 Sep [cited 2019 Jun 6];28(5):w822–31. Available from: http://www.ncbi.nlm.nih.gov/pubmed/19635784
26. Atkins CY, Thomas TK, Lenaker D, Day GM, Hennessy TW, Meltzer MI. Cost-effectiveness of preventing dental caries and full mouth dental reconstructions among Alaska Native children in the Yukon-Kuskokwim delta region of Alaska. J Public Health Dent [Internet]. 2016 [cited 2019 Jun 6];76(3):228–40. Available from: http://www.ncbi.nlm.nih.gov/pubmed/26990678
